# MZF1 and SCAND1 Reciprocally Regulate *CDC37* Gene Expression in Prostate Cancer

**DOI:** 10.3390/cancers11060792

**Published:** 2019-06-08

**Authors:** Takanori Eguchi, Thomas L. Prince, Manh Tien Tran, Chiharu Sogawa, Benjamin J. Lang, Stuart K. Calderwood

**Affiliations:** 1 Division of Molecular and Cellular Biology, Department of Radiation Oncology, Beth Israel Deaconess Medical Center, Harvard Medical School, 330 Brookline Avenue/CLS 610, Boston, MA 02115, USA; tprince@geisinger.edu (T.L.P.); bjlang@bidmc.harvard.edu (B.J.L.); 2 Department of Dental Pharmacology, Graduate School of Medicine, Dentistry and Pharmaceutical Sciences, Okayama University, Okayama 700-8558, Japan; trantienmanh1508@gmail.com (M.T.T.); caoki@md.okayama-u.ac.jp (C.S.); 3 Departments of Urology and Functional Genomics, Geisinger Clinic, Danville, PA 17822, USA

**Keywords:** SCAN zinc finger, SCAND1, CDC37, MZF1, prostate cancer

## Abstract

Cell division control 37 (CDC37) increases the stability of heat shock protein 90 (HSP90) client proteins and is thus essential for numerous intracellular oncogenic signaling pathways, playing a key role in prostate oncogenesis. Notably, elevated expression of CDC37 was found in prostate cancer cells, although the regulatory mechanisms through which CDC37 expression becomes increased are unknown. Here we show both positive and negative regulation of *CDC37* gene transcription by two members of the SREZBP-CTfin51-AW1-Number 18 cDNA (SCAN) transcription factor family—MZF1 and SCAND1, respectively. Consensus DNA-binding motifs for myeloid zinc finger 1 (MZF1/ZSCAN6) were abundant in the *CDC37* promoter region. MZF1 became bound to these regulatory sites and *trans*-activated the *CDC37* gene whereas MZF1 depletion decreased CDC37 transcription and reduced the tumorigenesis of prostate cancer cells. On the other hand, SCAND1, a zinc fingerless SCAN box protein that potentially inhibits MZF1, accumulated at MZF1-binding sites in the *CDC37* gene, negatively regulated the *CDC37* gene and inhibited tumorigenesis. SCAND1 was abundantly expressed in normal prostate cells but was reduced in prostate cancer cells, suggesting a potential tumor suppressor role of SCAND1 in prostate cancer. These findings indicate that CDC37, a crucial protein in prostate cancer progression, is regulated reciprocally by MZF1 and SCAND1.

## 1. Introduction

The cell division control 37 (CDC37) protein plays a fundamental role in chaperoning the protein kinase family and participates in cancer by maintaining the activity of protein kinases involved in cell proliferation and transformation. These include tyrosine kinases such as Src [1], serine/threonine kinases in the Raf-ERK pathway [2], Akt [3], the inhibitor of NF-κB kinase (IKK) [4], and cyclin-dependent kinase 4 (CDK4) [5,6,7]. CDC37 functions primarily in a complex with heat shock protein 90 (HSP90) to mediate the three-dimensional folding and structural integrity of client protein kinases [1,8,9]. CDC37 is particularly significant in prostate cancer as its overexpression leads to prostate carcinogenesis in transgenic mice [10,11,12]. It has been suggested that the high levels of oncogenic proteins present in most cancers make them dependent on molecular chaperones, a state referred to as “chaperone addiction” [13,14]. Thus, because of their large protein clienteles, the CDC37–HSP90 axis offers a critical target for inactivating multiple oncogenic pathways [13,14]. Consequently, the inhibition of HSP90 in cancer is currently a major area of research [14,15]. However, less is known regarding the regulation of CDC37 expression in cancer. Thus, we have addressed this deficiency in this study.

Examination of the *CDC37* 5′ upstream region and introns indicated multiple consensus sequences that are potentially bound by the transcription factor myeloid zinc finger 1 (MZF1, also known as ZSCAN6, ZNF42, ZFP98), consistent with a previous study [16]. We recently reported that the frequent amplification of MZF1 was observed in human cancers, further suggesting an oncogenic role for this factor [17]. Early studies showed that MZF1 might play roles in stemness and in the differentiation of hematopoietic stem cells and indicated its involvement in myeloma [18]. MZF1 was shown to participate in the malignant properties of several major solid tumors, including breast [19,20], lung [21], liver [22], head and neck [23], skin [24], endometrium [25], colorectal, and cervical cancers [26]. MZF1 protein structure is composed of an N-terminal SREZBP-CTfin51-AW1-Number 18 cDNA (SCAN) domain, a linker region, and a C-terminal DNA binding domain that contains 13 zinc finger (ZF) motifs and is a member of the SCAN zinc finger (SCAN-ZF) family [17,27]. The SCAN domain is a leucine-rich oligomerization domain and is highly conserved in the SCAN-ZF family, which is composed of more than 50 family members [28,29]. Zinc fingerless SCAN domain-only proteins also exist [27]. SCAND1 is a SCAN domain-only protein that has been shown to bind MZF1 and other SCAN-ZF family members. SCAN domain-only proteins have been suspected to be suppressors of intact SCAN-ZF transcriptional activity [27,28,30]. This possibility, however, has remained experimentally untested. Furthermore, we have tested the hypothesis that the relationship between MZF1 and SCAND1 regulates CDC37 expression and thereby cancer growth.

In this report we characterized the *CDC37* gene promoter and determined that MZF1 indeed increases CDC37 expression, while SCAND1 represses CDC37 expression. Our findings provide insight into how MZF1-driven CDC37 expression promotes cancer progression and how SCAND1 functions as a potential tumor suppressor by repressing CDC37.

## 2. Results

### 2.1. Elevated Expression of CDC37 Is Caused by MZF1 in Prostate Cancer

**Figure 1:**
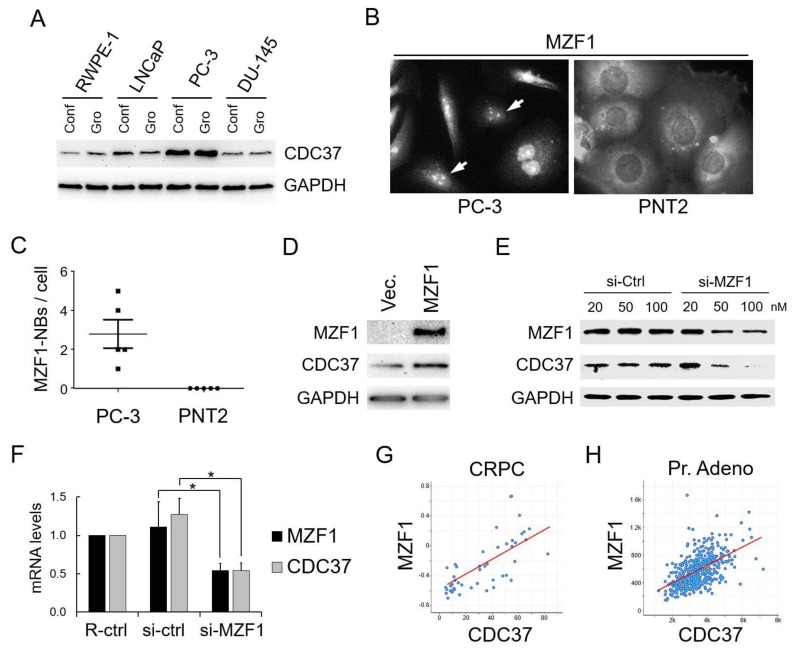
Elevated expression of cell division control 37 (CDC37) is caused by MZF1 in prostate cancer. (**A**) Western blot showing CDC37 in prostate cancer cells. Cell lysates were prepared from confluent (Conf) and growing (Gro) RWPE-1, LNCaP, PC-3, and DU-145 cells. (**B**) Immunocytochemistry showing MZF1 expression and localization in PC-3 and PNT2 cells. Arrows indicate nuclear bodies of MZF1 (MZF1-NBs) seen in PC-3 but not in PNT2. (**C**) The number of MZF1-NBs per cells. (**D**) Western blot showing CDC37 altered by overexpressed MZF1 in DU-145. (**E**) Western blot showing CDC37 altered by the depletion of MZF1 in DU-145 cells. (**F**) mRNA levels of CDC37 and MZF1 altered by the depletion of MZF1. The MZF1-targeting siRNA mixture (A + B + C, 10 nM each) or a non-silencing control (si-ctrl) was transfected into PC-3 cells. RPL32, internal control. R-ctrl, reagent only control. * *p* < 0.05, *n* = 3. Similar results were obtained from three independent experiments. (**G**) Co-expression of MZF1 and CDC37 in patient samples of castration-resistant prostate cancer (CRPC). *N* = 114, Spearman correlation score 0.78, *p* = 2.60 × 10^−11^. (**H**) Co-expression of MZF1 and CDC37 in patient samples of prostate adenocarcinoma (Pr. Adeno). *N* = 494, Spearman correlation score 0.57, *p* = 7.74 × 10^−44^.

We first compared the expression levels of CDC37 between prostate cancer cell lines DU-145 and LNCaP, a castration-resistant prostate cancer (CRPC, also known as neuroendocrine prostate cancer (NEPC) cell line PC-3), and a normal prostate cell line (RWPE-1). CDC37 levels in PC-3 cells were higher than those of the other prostate cancer cells and normal prostate cells in both confluent and growing conditions (Figure 1A). CDC37 levels in the prostate cancer cells (PC-3, LNCaP, and DU-145) were higher than those of the normal cell line in confluent conditions (Figure 1A).

Although there are several possible mechanisms for the elevation in CDC37 expression in prostate cancer, in the present study we focused on transcriptional regulation as the most immediate level of control. To determine potential *cis*-acting regulatory elements in the gene, we analyzed the DNA sequence between −3.6 kbp and +2 kbp from the *CDC37* transcription start site (TSS) and found numerous consensus MZF1 binding sequences (Supplementary Materials Figure S1).

To query the increased CDC37 level observed in PC-3 cells shown in Figure 1A, we next examined MZF1 expression and localization. MZF1 localized in nuclei in PC-3 but in the cytoplasm in the normal prostate cell line PNT2 (Figure 1B), suggesting that both the expression and nuclear localization of MZF1 could be involved in the increased CDC37 level in PC-3 cells. Additionally, nuclear bodies of MZF1 (designated as MZF1-NBs inasmuch as MZF1 oligomers could form nuclear bodies) were seen in PC-3 whereas cytoplasmic retention of MZF1 was seen in prostatic normal PNT2 cells, although the significance of the MZF1-NBs is currently not known (Figure 1B,C). Moreover, MZF1 overexpression led to increases in CDC37 levels in PC-3 cells (Figure 1D), while siRNA-mediated knockdown of MZF1 lowered CDC37 mRNA and protein levels (Figure 1E,F). A role for MZF1 in CDC37 transcription was thus suspected.

To investigate the potential clinical significance of MZF1 regulation of CDC37, we next examined the co-expression correlation of MZF1 and CDC37 in prostate cancer patient-derived tumor samples. Co-expression correlation was found between MZF1 and CDC37 in CRPC patient samples (Spearman’s rank correlation score: 0.78) and in prostate adenocarcinoma patient samples (Spearman’s correlation: 0.41) (Figure 1G,H). We next examined MZF1 and CDC37 expression correlation with the prognosis of prostate cancer patients. High expression levels of MZF1 (*p* = 0.0287) and CDC37 (*p* = 0.0182) were correlated with poor prognosis of patients suffering from prostate cancer and showed a higher correlation than that of PSA (*p* = 0.154)—named after prostate-specific antigen—which is currently used for prostate cancer screening (Table 1).

These data indicated MZF1 to be a potential causal transcription factor for the elevated expression of CDC37 in prostate cancer.

### 2.2. SCAN Zinc Finger Protein MZF1 Directly Trans-Activates the CDC37 Gene

**Figure 2:**
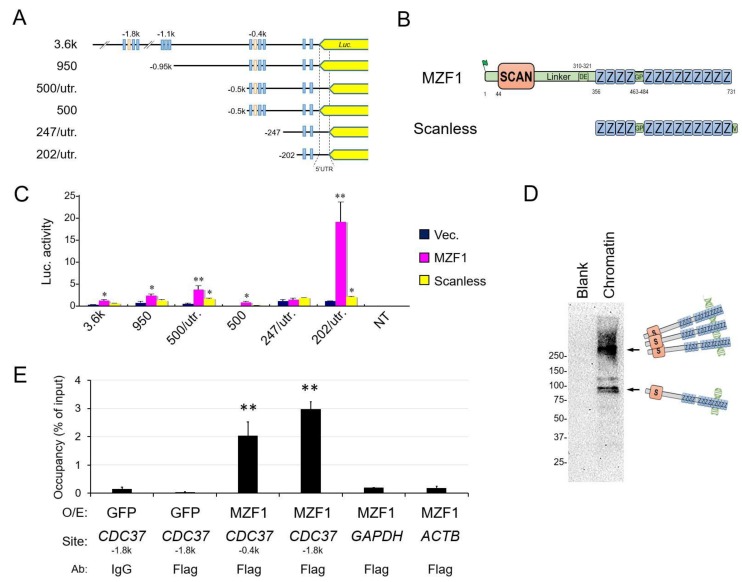
SCAN zinc finger MZF1 directly *trans*-activates the *CDC37* gene. (**A**) Schemes of the *CDC37* promoter–reporter constructs. Truncated mutants of *CDC37* promoter were connected with the *luciferase* (*Luc*) gene. Blue box, MZF1 binding site. Orange box, heat shock element (HSE). 5′UTR, 5′ untranslated region. (**B**) The secondary structures of native MZF1 and the SCANless construct (zinc finger domain alone). S, SCAN box. Z, zinc finger motif. DE, aspartic acid- and glutamic acid-rich region. GP, glycine- and proline-rich region. MZF1 was overexpressed with an N-terminal Flag-tag. The SCANless construct was overexpressed with a C-terminal V5-tag. (**C**) Luciferase activities from the truncated CDC37 promoters controlled by MZF1 and the SCANless construct. Plasmid constructs shown in panels A and B were co-transfected into DU-145 cells. Vec., empty vector control. *n* = 3, * *p* < 0.05, ** *p* < 0.01 (vs. Vec.). (**D**) Chromatin Western blotting of MZF1 overexpressed in DU-145. Crosslinked chromatin was prepared from DU-145 overexpressed with MZF1. (**E**) ChIP-qPCR analysis showing MZF1 occupancy of *CDC37*. Chromatin was prepared from DU-145 overexpressed with Flag-MZF1 or GFP (control) and immunoprecipitated using anti-Flag antibody beads or the control, IgG. Co-immunoprecipitated DNA was analyzed by qPCR for *CDC37* (−1.8k or −0.4k regions) or control regions in *GAPDH* or *ACTB*. *N* = 3, ** *p* < 0.01 (vs. IgG control). Similar results were obtained from three independent experiments.

We next queried whether the SCAN zinc finger MZF1 could directly *trans*-regulate the *CDC37* gene through direct binding to *cis*-elements (MZF1 binding sequences) abundantly found in the *CDC37* 5′ upstream region (Supplementary Materials Figure S1). We examined the activities of *CDC37* promoter-driven luciferase reporter constructs containing different deletion mutants of the 5′ region and examined potential *trans*-regulation by MZF1 and its SCANless truncation constructs of the factor (Figure 2A,B). The full-length native MZF1 markedly increased *CDC37* promoter activities of the 3.6k, 950, 500/utr, 500, and 202/utr constructs (Figure 2C). The SCANless mutant slightly increased *CDC37* promoter activities (of 500/utr, 202/utr) but the transcriptional activity was greatly reduced compared with SCAN-containing native MZF1. These data indicate that the SCAN oligomerization domain is essential for the transcriptional activity of MZF1. It is notable that the 202/utr reporter was more potent in *trans*-activation compared to other constructs, suggesting the presence of potential inhibitory motifs upstream of the 202 sequence. We next examined whether the overexpressed MZF1 (with Flag-tag) could directly bind to *cis*-elements in the genomic *CDC37* promoter region. MZF1 was detected by immunoblot in the crosslinked chromatin fraction and potential post-translational modification forms (upshift) and potential oligomers were detected in the chromatin (Figure 2D). The overexpressed MZF1 occupied genomic *CDC37* promoter regions (−0.4k and −1.8k regions that contain MZF1-binding sites), further indicating that the *CDC37* gene is regulated by MZF1 binding to these MZF1 binding sites in prostate cancer, as suggested by chromatin immunoprecipitation (ChIP) assay (Figure 2E).

These data indicate that MZF1 directly *trans*-activates the *CDC37* gene through direct binding to the *CDC37* promoter region and that SCAN domain-mediated oligomerization is essential for the full transcriptional activity of MZF1.

### 2.3. The Zinc Fingerless SCAND1 Factor Suppresses CDC37 Gene and Tumorigenesis of Prostate Cancer

**Figure 3:**
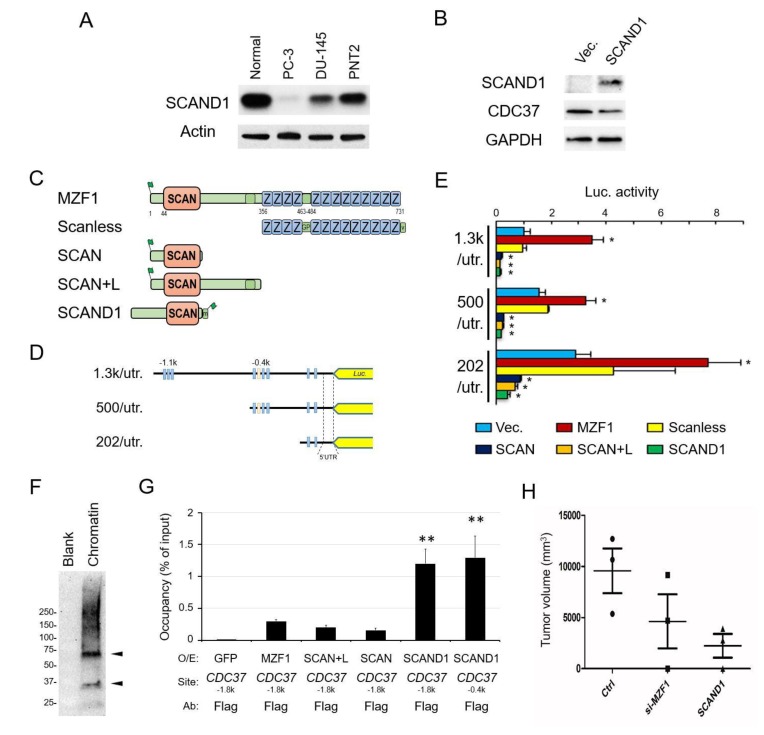
Zinc fingerless SCAND1 suppresses the *CDC37* gene and tumorigenesis of prostate cancer. (**A**) Western blot showing SCAND1 levels in normal prostate cells, PC-3, DU-145, and PNT2. (**B**) Western blot showing CDC37 lowered by SCAND1 overexpression. (**C**) Schemes of overexpression constructs. (**D**) Schemes of truncated *CDC37* promoters fused with the luciferase reporter gene. (**E**) Luciferase activities from the truncated *CDC37* promoters controlled by SCAND1 and truncated mutants of MZF1. Plasmid constructs shown in panels C and D were co-transfected into PC-3 cells. Vec., empty vector control. *n* = 3, * *p* < 0.05 (vs. Vec.). (**F**) Chromatin Western blot showing SCAND1 overexpressed in DU-145. Crosslinked chromatin was prepared from DU-145 overexpressed with SCAND1. (**G**) ChIP-qPCR analysis showing SCAND1 occupancy of CDC37. Chromatin was prepared from DU-145 overexpressed with SCAND1, MZF1, SCAN, SCAN + L, or GFP (control) and immunoprecipitated using anti-Flag antibody beads. Co-immunoprecipitated DNA was analyzed by qPCR for CDC37 (−1.8k or −0.4k regions). *N* = 3, ** *p* < 0.01 (vs. IgG control). For Figure 3A–G, similar data were obtained from three independent experiments. (**H**) Tumorigenicity of PC-3 cells lowered by SCAND1 and the depletion of MZF1. The SCAND1-overexpression plasmid, MZF1-targeted siRNA, and control GFP plasmid were transfected into PC-3 cells, which were then xenografted to immunocompromised mice subcutaneously.

The SCAN-ZF family consists of more than 50 members and contains a few potentially inhibitory zinc fingerless members such as SCAND1. We hypothesized that SCAND1 could play a key role in limiting functions of numerous SCAN-ZF proteins such as MZF1 through hetero-oligomerization. Notably, SCAND1 is highly expressed in normal prostate tissue compared to other tissues (http://biogps.org/#goto=genereport&id=51282). We found SCAND1 to be expressed in normal prostate cells while its levels were reduced in prostate cancer PC-3 and DU-145 cells (Figure 3A), suggesting that SCAND1 expression declines along with prostate oncogenesis. Overexpression of SCAND1 led to lowered CDC37 levels in PC-3 cells (Figure 3B).

To determine whether SCAND1 and a SCAN-only construct derived from MZF1 could regulate *CDC37* promoter constructs, we next carried out co-transfection and reporter assays. SCAND1 and SCAN-only constructs (SCAN and SCAN + linker) derived from the truncation of MZF1 significantly repressed *CDC37* promoter activities (1.3k/utr, 500/utr, 202/utr) in PC-3 cells (Figure 3C–E). We also confirmed that the native MZF1 protein activated the *CDC37* promoter whereas the SCANless mutant had little effect.

We next hypothesized that SCAND1, although lacking a discernible DNA-binding domain, might be able to associate with genomic CDC37 promoter regions. Overexpressed SCAND1 was detected on chromatin in PC-3 as a monomer of the expected molecular weight. We also observed potential dimer and higher molecular weight forms in the crosslinked chromatin, suggesting the higher oligomerization of SCAND1 on chromatin, which might be consistent with a powerful repressor role (Figure 3F). The overexpressed SCAND1 (with Flag-tag) occupied the *CDC37* promoter region (−1.8k and −0.4k regions, which we showed to contain MZF1-binding sites) (Figure 3G). The SCAND1 occupancy of the CDC37 regulatory site (−1.8k) was higher in magnitude than that observed with SCAN-only constructs of MZF1, as suggested by the increased enrichment in the ChIP assay. We consistently observed that overexpressed SCAND1, as well as MZF1, was found in the insoluble chromatin-containing fraction, whereas overexpressed SCAN domain (SCAN, SCAN + L) constructs tended to be found in the soluble fraction of the cell lysate (Supplementary Materials Figure S2).

These data indicate that SCAND1 could repress the *CDC37* gene powerfully through association with DNA sequences that we showed to bind MZF1. Therefore, our data indicate that while MZF1 positively regulates CDC37, SCAND1 negatively regulates transcription, a finding which could be crucial in mechanisms of prostate cancer progression.

We therefore hypothesized that, in this context, *MZF1* has an oncogenic influence while *SCAND1* may suppress tumorigenicity. To elucidate this possibility, we asked whether tumor initiation by prostate cancer PC-3 cells could be suppressed by SCAND1 expression and by the depletion of MZF1. Indeed, we observed that SCAND1 overexpression suppressed tumorigenicity, while the depletion of MZF1 by siRNA lowered tumorigenicity in PC-3 cells (Figure 3H).

## 3. Discussion

Our data suggest a novel mechanism for prostate cancer regulation by MZF1 (Figure 1). CDC37 is a crucial molecule in prostate cancer growth through its fostering of oncogenic kinases and our data strongly indicate that the chaperone is upregulated by MZF1. We showed that MZF1 binds to sites in the *CDC37* promoter and strongly activates transcription; moreover, MZF1 expression is tightly correlated with CDC37 levels in clinical prostate cancer (Figure 1 and Figure 2, Table 1). The ability of an endogenous inhibitor, the zinc fingerless SCAND1 factor, to bind the promoter and repress the transcription of MZF1 suggests a potential mechanism for prostate tumor suppression (Figure 3). These data suggest mechanisms whereby members of the SCAN transcription factor family can fine-tune prostate cancer growth by up or downregulating CDC37 transcription and thus decide the outcome of prostate tumorigenesis. It is likely that other MZF1 targets important in tumorigenesis may be regulated in a similar way [17], including oncogenes and pro-tumorigenic genes such as *MYC* [21], *NCAD* [31], *YAP1* [32], *TGF-beta* [20], *CTSB/L* [33], *MMP-14* [34], *FOXM1* [35], *CK17* [36], *PAX2* [25], *PRAME* [24], *DR5* [37], *AXL* [26,38], *PKC-alpha* [39,40], *CD34*, and *c-Myb* [18]. Besides, MZF1 might play key roles also in tumor microenvironment such as mesenchymal stem cell differentiation into cancer-associated fibroblasts [20]. However, MZF1 has also been shown to activate tumor suppressor genes such as *FPN* [41], *NFKBIA* [42], and *SMAD4* [43]. In a more complex manner, MZF1 also plays a transcriptional repressor role for pro-tumorigenic genes such as *MMP-2* [44] and *IGF-IR* [45]. Nevertheless, our data do not exactly define the mechanism through which SCAND1 represses MZF1 activity, although direct co-occupation of the *CDC37* promoter appears to be involved (Figure 3). MZF1 was shown to bind DNA as a homodimer or heterodimer with other SCAN domain proteins and recruit the chromatin remodeling protein mDomino [46]. Our data pinpoint the importance of the SCAN domain, as the SCANless construct of MZF1 showed much reduced transcriptional activity compared to the powerful activity of the native MZF1 (Figure 3E). The SCAN domain is leucine-rich and mediates oligomer formation [17]. We found that MZF1 could form a higher order structure, potentially an oligomer on chromatin (Figure 2D). Therefore, it was indicated that the SCAN domain of MZF1 is required for its oligomerization and thus for the full activity for *trans*-activation. We also observed up-shift bands of MZF1 as potential post-translational modifications required for MZF1 activity (Figure 2D). It has been reported that MZF1 could be phosphorylated by PAK4 in a SUMO-directed manner [47] and by CK2 [31] and ERK-1/2 [36]. It was also reported that the loss of the nuclear pool of ubiquitin ligase CHIP/STUB1 unleashes the MZF1-cathepsin pro-oncogenic program [33]. Thus, upstream regulatory signals that modify specific amino acid residues of MZF1 have been recently clarified. However, little is currently known regarding the mechanisms whereby SCAND1 could repress transcription, although our data indicate that this may be associated with the CDC37 promoter in the oligomeric form at the site of MZF1 binding (Figure 3).

Our studies on CDC37 are ultimately aimed at targeting this factor in cancer as an alternative to HSP90. Numerous HSP90 inhibitors have been developed to target the abundant HSP90, which plays versatile oncogenic roles in many types of cancer [48]. Moreover, extracellular HSP90 and extracellular vesicle-associated HSP90 have been recently shown to play pro-tumorigenic roles [49,50,51,52,53]. However, it has been difficult so far to employ HSP90 inhibitors at effective doses due to normal tissue toxicity. Depletion of CDC37 reduced prostate cancer cell growth and attenuated the MEK-ERK signaling pathway and the PI3K-Akt signaling pathway [11]. Moreover, overexpression of CDC37 triggers prostatic tumorigenesis [10]. Our studies therefore suggest that further study of MZF1 and SCAND1 activities in regulating CDC37 transcription may identify a new therapeutic angle to target the cohort of oncogenic signaling processes dependent upon the HSP90–CDC37 complex.

## 4. Materials and Methods

### 4.1. Cell Culture

PC-3, DU-145, LNCaP, and RWPE-1 cells were obtained from ATCC. PC-3 was cultured in F-12K or RPMI 1640 medium containing 10% fetal bovine serum (FBS). DU-145 was cultured in DMEM containing 10% FBS. LNCaP was cultured in RPMI 1640 medium containing 10% FBS. RWPE-1 was cultured in Keratinocyte Serum-Free Medium (Thermo Fisher Scientific, Waltham, MA, USA) supplemented with bovine pituitary extract (BPE) and human recombinant epidermal growth factor (hEGF). PNT2 was obtained from Sigma and cultured in RPMI 1640 medium supplemented with 2 mM glutamine and 10% FBS. Normal human prostate epithelial cells were purchased from Lonza (Basel, Switzerland) and cultured in Prostate Epithelial Cell Basal Medium (Lonza) supplemented with BPE, hydrocortisone, hEGF, epinephrine, transferrin, insulin, retinoic acid, triiodothyronine, and GA-1000.

### 4.2. Molecular Cloning

Genomic DNA was isolated from a buccal cell swab of a deidentified white male. The 3.6 kbp human *CDC37* promoter region was PCR-amplified and subcloned into a pGL3-luciferase vector (Promega, Madison, WI, USA) using *Kpn*I and *Bam*HI. Once the pGL3-3.6 kbp CDC37promo vector was made, the 1.3 kbp, 950 kbp, 500 bp, 247 bp, and 202 bp fragments with and without the 116 bp 5′UTR were similarly subcloned into pGL3 using the same restriction sites as above. Each entire promoter fragment clone was Sanger sequenced. The complete 3.6 kbp *CDC37* promoter sequence was aligned with the human reference genome GRCh37.

Human MZF1 cDNA was subcloned from pOTB-MZF1 into pcDNA3-Flag vector via TOPO directional cloning (Thermo Fisher Scientific) and designated as pcDNA3/Flag-MZF1. For SCAN-only constructs, UGA stop codons were generated in the pcDNA/Flag-MZF1 via Quickchange mutagenesis and designated as pcDNA3/Flag-C125/SCAN and pcDNA3/Flag-C252/SCAN + L. For SCANless constructs, the cDNA of the MZF1 zinc finger domain was amplified via PCR and subcloned into pcDNA/V5 vector using TOPO directional cloning and designated as pcDNA/MZF1-V5/N252 (SCANless). Human SCAND1 (NM_033630) open reading frame clone with Myc-DDK C-terminal tag (RC200079) was purchased (Origene, Rockville, MD, USA) and designated as pCMV6-ScanD1-myc-Flag. pCMV-EGFP (NEPA Gene) was used as an overexpression control.

### 4.3. In Silico Analysis of Promoters and Gene Bodies

Sequences of promoter regions and gene bodies of human *CDC37, HSP90AA1*, and *HSP90AB1* were obtained from the Eukaryotic Promoter Database [54]. Binding sites for MZF1 were predicted using PROMO [55,56].

### 4.4. Luciferase Assay

Transient transfection and luciferase assays were performed as previously described [57]. Cells were cultured in 96-well plates. A plasmid (25 ng reporter, 100 ng effector) was transfected with 0.4 µL FuGENE HD (Roche, Basel, Switzerland) per well at a cell confluence level of 50–70%. The medium was changed at 16–20 h after transfection. At 40–48 h after transfection, 70 µL of the medium was aspirated, then 30 µL of Bright-Glo reagent (Promega) was added and mixed by pipetting. Cells were incubated for 5 min at 37 °C. The lysate (40 µL) was transferred to a 96-well white plate for the measurement of luminescence.

### 4.5. siRNA

The siRNA was designed based on the siRNA design method of JBioS (Japan Bio Services, Saitama, Japan). An RNA duplex of 19 bp plus TT-3′ overhangs in each strand was synthesized by Nippon Gene (Tokyo, Japan). For electroporation-transfection, a total of 40 pmol siRNA was transfected to 5 × 10^5^ cells. For reagent-transfection, cells were transfected with siRNA at final concentrations of 20–100 nM. Non-targeting siRNA was purchased from Nippon Gene (Tokyo, Japan). The designed sequences of siRNA are listed in Supplementary Materials Table S1.

### 4.6. Transfection

For ChIP assay and tumorigenesis assay, electroporation-mediated transfection was performed as described previously [50,58]. To optimize electroporation for each cell type, cells (1 × 10^5^ to 1 × 10^6^ cells), plasmid DNA (2 to 10 µg total) or siRNA (40 pmol total), and serum-free medium were mixed to 100 µL total in a green cuvette with a 1 mm gap (NepaGene, Ichikawa, Tokyo) and placed in an NEPA21 Super Electroporator (NepaGene). The poring pulse was optimized between 100 V and 300 V for 2.5 or 5.0 ms pulse length twice with 50 ms intervals between the pulses and 10% decay rate with + polarity. The transfer pulse condition was five pulses at 20 V for 50 ms pulse length with 50 ms intervals between the pulses and 40% decay rate with +/− polarity. After electroporation, cells were recovered in serum-containing media. PC-3 cells (5 × 10^5^ cells) were electroporation-transfected with 10 µg plasmid DNA or 40 pmol siRNA with a poring pulse at 175 V for 2.5 ms pulse length twice.

For the overexpression ChIP assay, DU-145 cells (5 × 10^6^ cells) were transfected with 15 µg of pcDNA/Flag-MZF1 or pCMV-GFP with a poring pulse at 175 V for 5 ms pulse length twice, cultured for 4 days in a 15 cm dish, and then used for ChIP assay.

For Western blot analysis with overexpression of MZF1 and SCAND1, PC-3 cells were transfected with pcDNA3.1(–), pcDNA/Flag-MZF1, and pCMV6-SCAND1-myc-Flag using FuGENE6 (Roche).

For qRT-PCR after MZF1 depletion, PC-3 cells were transfected with a mixture of MZF1-siRNA A, B, and C (SR305183, OriGene, Rockville, MD) or non-silencing siRNA (SR30004, OriGene) using Lipofectamine RNAi max (Thermo Fisher Scientific).

For the depletion of MZF1 and Western blotting, DU-145 cells cultured in a 6-well plate were transfected with siRNA hMZF1-all-NM_003422-53 using Lipofectamine RNAi Max. The medium was replaced with a fresh one at 24 h after transfection. Cells were lysed at 48 h after transfection.

### 4.7. Chromatin

For protein overexpression and ChIP assay, DU-145 cells (5 × 10^6^ cells) were electroporation-transfected with pCMV6-ScanD1-myc-Flag, pcDNA3/Flag-MZF1, pcDNA3/Flag-SCAN, pcDNA/Flag-SCAN + L, and pCMV-GFP and then cultured for 3 days in 150 mm dishes. ChIP was conducted using a SimpleChIP enzymatic chromatin IP kit with magnetic beads (Cell Signaling Technology). Briefly, proteins/DNA were crosslinked with formaldehyde. For optimization, nuclear fractions were treated with MNase (0, 0.25, 0.5, 0.75, and 1 µL per reaction) and subsequent ultra-sonication at high and low power using an Ultrasonic Homogenizer Smurt NR-50M (Microtec Nition), and then analyzed within 2% agarose gel electrophoresis. After the optimization, the nuclear fractions were sheared with MNase (0.75 µL per reaction) and ultra-sonication at high power and then centrifuged at 10,000× *g* for 10 min at 4 °C. The supernatants were used as crosslinked chromatin fractions. Overexpressed proteins in the chromatin fraction were analyzed by Western blotting using anti-MZF1 antibody (Assay Biotechnology, Fremont, CA, USA) and anti-SCAND1 antibody (ab64828, Abcam, Cambridge, UK). Anti-Flag M2 magnetic beads (Sigma, St. Louis, MO, USA) were used for ChIP. DNA was purified from the eluate and from 1% and 10% input chromatin using a QIA-Quick DNA purification kit (Qiagen, Hilden, Germany) and eluted within 50 μL of ddH_2_O. The purified DNA (3–5 μL) was used for ChIP-qPCR. Amplification specificity and efficiency were confirmed with melting curve analysis, slope analysis, and agarose gel electrophoresis. Primers are listed in Supplementary Materials Table S2.

### 4.8. Tumorigenesis

The efforts of all animal experiments were made to minimize suffering. The studies were carried out in strict accordance with the recommendations in the Guide for the Care and Use of Laboratory Animals of the Japanese Pharmacological Society. The protocol was approved by the Animal Care and Use Committee, Okayama University (Permit Number: OKU-2016219). All animals were held under specific pathogen-free conditions. PC-3 cells (5 × 10^5^) were transfected with 40 pmol MZF1-targeted siRNA, 10 µg pCMV6-ScanD1-myc-Flag, or 10 µg pCMV-EGFP via electroporation, cultured for 5 days, and then 1 × 10^6^ cells were subcutaneously injected into the back of a 6–7 year old CB17/IcrJcl-Pkrdc^scid^ (SCID) mouse (CLEA, Japan, Tokyo). Tumor volumes were measured at day 41 post-injection. The major axis (a) and minor axis (b) of tumors were measured with a caliper. The tumors were deemed to be ellipsoid and the volumes were calculated as follows: tumor volume (V) ≒ 4πab^2^/3.

### 4.9. Western Blot

To compare CDC37 levels among several types of cells, cells were cultured to reach confluence in a 6 cm dish or sparsely grown in a 10 cm dish. For endogenous SCAND1, protein samples were collected when cells reached sub-confluence. At day 2 after medium replacement, cells were washed with phosphate buffered saline (PBS) and lysed within CelLytic M (Sigma) for 15 min with gentle shaking. Lysates were collected and centrifuged at 14,000× *g* for 15 min. Protein concentration was measured using BCA protein assay (Thermo Fisher Scientific). Protein samples (10–40 µg: equal amount) were loaded to SDS-PAGE. Proteins were transferred to a polyviniylidine difluoride (PVDF) membrane with a semi-dry method. The membranes were blocked within 5% skimmed milk in Tris buffered saline with 0.1% Tween 20 (TBST) for 1 h. The membranes were incubated with primary antibodies overnight at 4 °C and with secondary antibodies for 1 h at room temperature (RT). The membranes were washed three times with TBST for 15 min after each antibody reaction. Chemiluminescence was detected with a ChemiDoc MP Imaging System (Bio-Rad, Hercules, CA, USA).

For crosslinked chromatin Western blotting, see the Section 4.7. For regular Western blotting after the overexpression of MZF1 and SCAND1, cells were transfected with pcDNA3/Flag-MZF1 or pcDNA3(-) using FuGENE6 (Roche) and cultured for 24 h. Cells were washed with ice-cold PBS twice and lysed within RIPA buffer supplemented with a protease phosphatase inhibitor cocktail (Thermo Fisher Scientific) at 4 °C for 15 min, and then collected. The lysed cells were homogenized 10 times using a 25 gauge needle attached to a 1 mL syringe and then incubated on ice for 30 min. The lysate was centrifuged at 12,000× *g* for 20 min at 4 °C to remove debris. The protein sample amounts (10–50 µg) were used for SDS-PAGE and semi-dry transfer.

For the depletion of MZF1, DU-145 cells cultured in a 6-well plate were transfected with siRNA hMZF1-all-NM_003422-53 using Lipofectamine RNAi Max following the manufacturer’s instruction. The medium was replaced with a fresh amount at 24 h after transfection. Cells were collected using trypsin and counted at 48 h after transfection. The cell lysate was prepared using RIPA buffer as described above. The equal amount of lysate (25 µg) was loaded to each lane for SDS-PAGE (4–20% TGX gel, Bio-Rad). The proteins were transferred to a PVDF membrane with the wet-transfer method at 40 V for 16 h on ice with a transfer buffer (25 mM Tris, 192 mM glycine, 10% methanol, and 0.05% SDS). The membrane was washed three times with TBST for 15 min and then blocked for 1 h within 5% skimmed milk. MZF1 and CDC37 were detected by Western blotting.

The antibodies used were anti-MZF1 (C10502, Assay Biotechnology, 1:500), anti-CDC37 (D11A3, Cell Signaling Technology, 1:1000), anti-FLAG (Clone M2, Sigma), anti-SCAND1 (ab64828, Abcam, Cambridge, UK), and HRP-conjugated anti-GAPDH antibody (Clone 5A12, Fujifilm Wako, 1:5000).

### 4.10. Immunocytochemistry

Immunocytochemistry was performed as described [59]. Cells cultured in 4-well chamber slides were fixed with 4% (w/v) paraformaldehyde in PBS for 15 min. Cells were permeabilized with 0.2% Triton X-100 in PBS for 15 min. Cells were blocked in IHC/ICC blocking buffer high protein (eBioscience, San Diego, CA, USA) for 10 min and then reacted with anti-MZF1 antibody (1:50, C10502; Assay Biotechnology) and AlexaFluor488 secondary antibody (Thermo Fisher Scientific, 1:1000) in the blocking buffer. Cells were washed with PBS for 5 min twice between the steps. Cells were mounted with ProLong Gold AntiFade Reagent (Thermo Fisher Scientific). Fluorescence images were captured using an Axio Vision microscope equipped with an AxioCam MR3 (Zeiss, Oberkochen, Germany).

### 4.11. RT-qPCR

RT-qPCR was performed as previously described [59,60]. Total RNA was prepared using an RNeasy RNA purification system (Qiagen, Hilden, Germany) with *DNase* I treatment. cDNA was synthesized using an RT kit for RT-qPCR (Qiagen) with a mixture of oligo dT and random primers. The cDNA pool was diluted 5- to 20-fold. The cDNA standard with permissible slopes of PCR efficiencies was prepared by step dilution of the cDNA pool for the relative quantification of mRNA levels. In 20 µL of qPCR mix, 0.25 µM of each primer, 4–10 µL of diluted cDNA, and 10 µL SYBR green 2× Master Mix (Applied Biosystems, Waltham, MA) were added and reacted at 95 °C for 10 min, followed by 40 cycles at 95 °C for 15 s and 60 °C for 1 min. Dissociation curves with specific single-peaked PCR and proper amplification slopes for PCR efficiency were confirmed. LinRegPCR software was used for baseline fluorescence correction, and for the calculation of Cq values and PCR efficiency of amplicons [61]. Primer sequences are listed in Supplementary Materials Table S3.

### 4.12. Gene and Protein Expression in Clinical Samples

Co-expression of MZF1 and CDC37 was analyzed in TCGA in cBioPortal. Data sets of NEPC/CRPC (Trento/Cornell/Broad 2016, 114 samples) and prostate adenocarcinomas (TCGA, PanCancer Atlas; 494 patients/samples) were analyzed with Spearman’s rank correlation coefficient of co-expression.

For the correlation of protein expression with patient prognosis, Kaplan–Meier survival analysis was performed using 494 prostate cancer patient samples in the Human Protein Atlas Database.

### 4.13. Statistics

Data were expressed as the means ± SD unless otherwise specified. Comparisons of two were performed with an unpaired Student’s *t*-test.

## 5. Conclusions

In conclusion, it was demonstrated that pro-oncogenic MZF1 and its potential antagonist SCAND1 coordinately regulate the expression of CDC37, a factor that is essential for prostate cancer tumorigenesis. The SCAN domain is required for the full transcriptional activity of MZF1, whereas the SCAN-only factor SCAND1 is powerfully associated with chromatin and represses the tumorigenesis of prostate cancer cells.

## Figures and Tables

**Table 1:** Correlation between protein expression and prognosis of prostate cancer patients.

Protein Name	*p* Value	*N* (Total)	*N* (High)	*N* (Low)
MZF1	0.0287	494	99	395
CDC37	0.0182	494	198	296
PSA	0.154	494	389	105

## Supplementary Materials

The following are available online at https://www.mdpi.com/2072-6694/11/6/792/s1, Figure S1: Binding sites for MZF1 and HSF1 in the *CDC37* promoter, Figure S2: Western blotting showing overexpressed MZF1, its truncated mutants, and SCAND1, Table S1: List of siRNA sequences, Table S2: List of primers for ChIP-qPCR. Table S3: List of primers for RT-qPCR.

## Abbreviations

CDC37	Cell division control 37
CRPC	Castration-resistant prostate cancer
HSP	Heat shock protein
MZF1	Myeloid zinc finger 1
NEPC	Neuroendocrine prostate cancer
SCAN	SREZBP-CTfin51-AW1-Number 18 cDNA
