# Supplementary material for: MZF1 and SCAND1 Reciprocally Regulate CDC37 Gene Expression in Prostate Cancer
Source: Cancers (Basel). 2019 Jun 8;11(6):792. doi: 10.3390/cancers11060792 (PMC6627353; doi:10.3390/cancers11060792)
Supplement: Supplementary file 1 [file cancers-11-00792-s001.pdf]

Supplementary Materials: MZF1 and SCAND1 reciprocally regulate *CDC37* gene expression in prostate cancer

Taka Eguchi, Thomas L. Prince, Manh Tien Tran, Chiharu Sogawa, Benjamin J. Lang and Stuart K. Calderwood

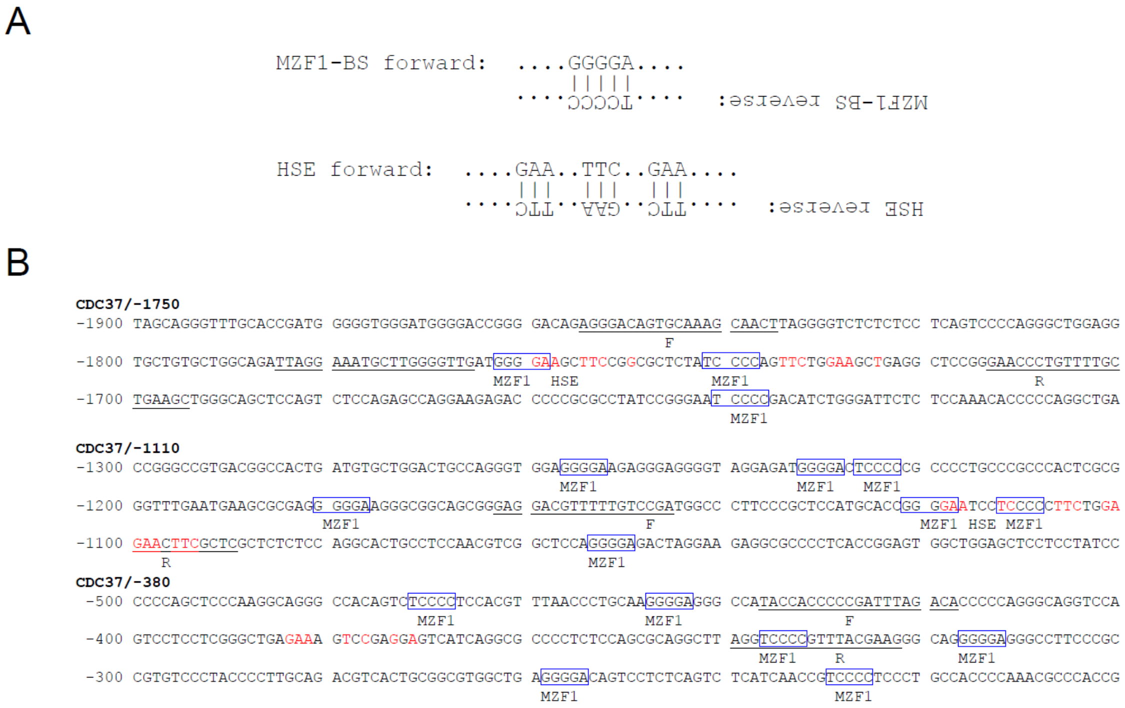

Figure S1. Binding sites for MZF1 and HSF1 in the *CDC37* promoter. (**A**) Consensus DNA sequences for MZF1 and heat shock elements (HSE). (**B**) Putative binding sites for MZF1 and HSF1 in *CDC37*. MZF1 binding sites were enclosed with blue boxes. Putative HSE were shown with red. The sequences detected by forwarding primers (F) and reverse primers (R) in ChIP-qPCR were underlined.

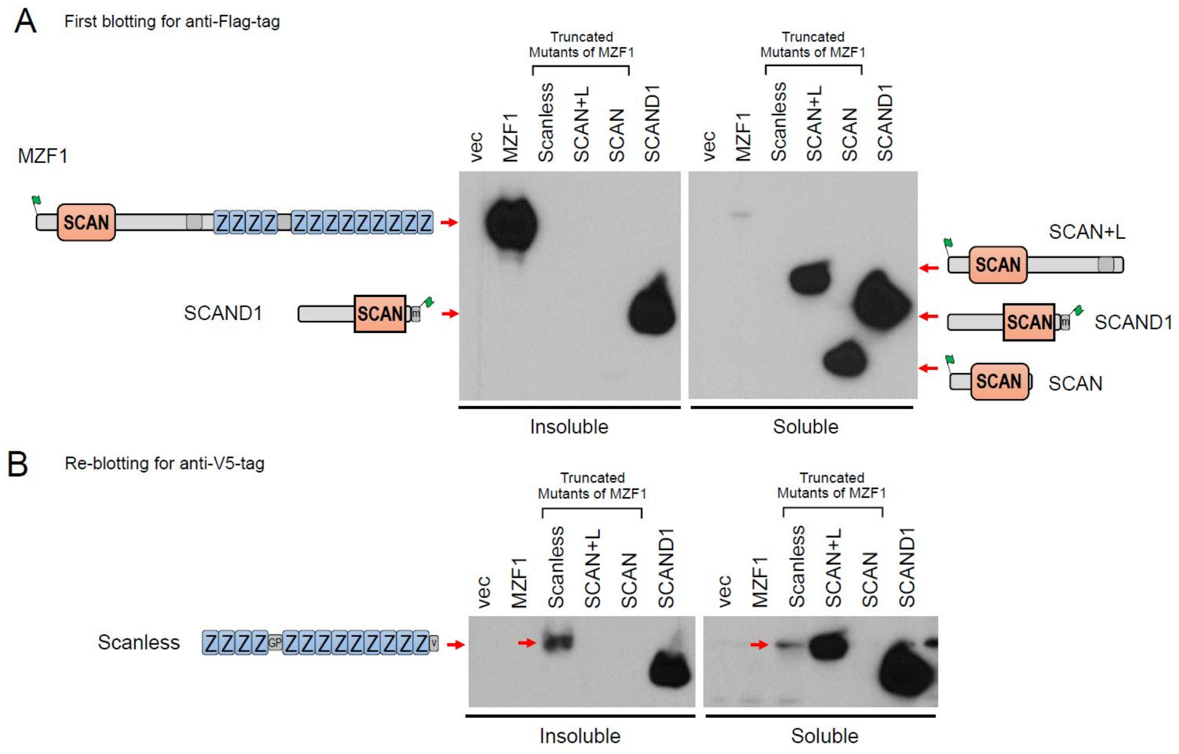

Figure S2. Western blotting showing overexpressed MZF1, its truncated mutants, and SCAND1. COS7 cells were transfected with plasmid constructs- Flag-MZF1, Scanless-V5, Flag-SCAN+L, Flag-SCAN, and SCAND1-Flag. The soluble cell lysate and the insoluble fraction containing chromatins were analyzed by western blotting using firstly anti-Flag tag antibody (**A**) and secondly anti-V5 tag antibody re-blotting (**B**). MZF1 and Scanless zinc-fingers-only constructs tended to be enriched in the insoluble fraction. The SCAN and SCAN+L tended to be found in the soluble fraction. SCAND1 was found in both insoluble and soluble fractions.

Table S1. List of siRNA sequences.

Name of siRNA	Sequence (5′ to 3′)
hMZF1-all-NM_003422-53 sense	ccaagccuuucuccauuuuTT
hMZF1-all-NM_003422-53 antisense	aaaauggagaaaggcuuggTT

Table S2. List of primers for ChIP-qPCR.

Name of primers	Sequence (5′ to 3′)
CDC37/-1750F/160bp	AGGGACAGTGCAAAGCAACT
CDC37/-1750R/160bp	GCTTCAGCAAAACAGGGTTC
CDC37/-380F/115bp	TACCACCCCCGATTTAGACA
CDC37/HSE/-380R/115bp	CTTCGTAAACGGGGACCTAA

Table S3. List of primers for qRT-PCR.

CDC37-h1030F/1693	TCCAGAAGTGCTTCGATGTG
CDC37-h1140R/1693	AGAGGCCAGAGTCAATGCAG
MZF1-h785F/2620	TGCAGGTGAAAGAGGAGTCA
MZF1-h939R/2620	AGTCTTGCTGTGGGGAAAGA
RPL32 F	CAGGGTTCGTAGAAGATTCAAGGG
RPL32 R	CTTGGAAACATTGTGAGCGATC
